# Glomerular Disease and Cystic Kidney Disease: From Pathogenesis to Novel Therapeutic Approaches

**DOI:** 10.3390/biomedicines13102322

**Published:** 2025-09-23

**Authors:** Vassilis Filiopoulos

**Affiliations:** Department of Nephrology and Kidney Transplantation, School of Medicine, National and Kapodistrian University of Athens, General Hospital of Athens “Laiko”, 11527 Athens, Greece; vassilis.filiopoulos@hotmail.com

This Special Issue [1] focuses on glomerular disease, both primary and secondary, and cystic kidney disease, especially polycystic kidney disease, the most common hereditary kidney disease. For both of these entities, there has been notable progress in unravelling the complex pathophysiology, and various potential therapeutic targets are available, for which novel treatments are being developed or repurposed.

The purpose of this Editorial is to provide a brief overview of the articles published in this Special Issue and their contribution to resolving knowledge gaps in this broad and complex field. Our aim is to further encourage the reader to explore them, rather than simply providing a summary of this Special Issue.

Glomerular disease describes a variety of relatively rare immune-mediated entities characterized by damage to the glomerular compartment of the kidney. Immune mechanisms of glomerular injury are particularly diverse and involve defects in innate and adaptive immunity, genetic factors, and unfavorable environmental conditions [2].

Podocytes are glomerular visceral epithelial cells that play a central role in glomerular diseases, especially those presenting with massive proteinuria, and contribute to crescent formation through the release of soluble factors [3]. Lianos et al. assessed whether constitutively expressed heme oxygenase (HO-1) regulated complement regulatory protein decay-accelerating factor (DAF, CD55) in cultured rat podocytes. The preliminary observations in this study indicated that in cultured rat podocytes, there is constitutive HO-1 and CD55 expression that can be increased through non-toxic heme concentrations. The regulatory effect of HO-1 on CD55 under conditions of podocyte exposure to heme remains to be examined and is highly relevant in conditions of systemic or intraglomerular hemolysis in which free heme can activate the complement cascade.

In a prospective observational study, Papasotiriou et al. investigated the effects of the mineralocorticoid receptor (MR) antagonist eplerenone in patients with biopsy-proven glomerulonephritis who had already been treated with ACEi or ARBs. Eplerenone showed a significant impact on proteinuria in those with baseline proteinuria above 1000 mg/24 h as an add-on treatment to ACEi or ARBs in patients with chronic glomerulonephritis with a favorable safety profile.

Panagakis et al. [4] performed a systematic review and meta-analysis to assess the influence of idiopathic membranous recurrence on graft survival, as well as to elucidate potential risk factors for recurrence and the role that treatment exerts on outcomes. The main conclusion of this systematic review was that the recurrence of idiopathic membranous nephropathy was not associated with an increased risk of graft loss independently of whether patients were treated with rituximab or not. Furthermore, patients who achieved remission had a significantly reduced risk of graft loss.

Autosomal dominant polycystic kidney disease (ADPKD) is the most common inherited kidney disorder worldwide and accounts for 5–10% of end-stage kidney disease (ESKD) cases in the US and Europe [5]. The mechanisms of cyst formation and growth are complex and largely inconclusive and include multiple aberrant processes, such as abnormal fluid secretion, cell proliferation and apoptosis and abnormal cilia function, and multiple signaling pathways [6]. Emerging evidence indicates that metabolic reprogramming plays a crucial role in ADPKD pathogenesis, including enhanced aerobic glycolysis, impaired fatty acid oxidation, glutamine dependence, and mitochondrial dysfunction [7]. Cao and Yu [8] provide a comprehensive review of the complex interactions between metabolic pathway alterations and key signaling cascades in ADPKD, in addition to exploring potential therapeutic approaches, both dietary and pharmaceutical, that may contribute to achieving the ultimate goal of slowing kidney disease progression in ADPKD patients in the future.

Gu et al. comprehensively review recent advancements in ADPKD organoid construction, particularly an induced pluripotent stem cell (iPSC)-derived model. Organoid models closely replicate the natural progression of diseases and can be used to study tissue development, organogenesis, and stem cell behavior in vitro. They hold significant potential for disease modeling and predicting personalized clinical outcomes [9]. Several laboratories have successfully established ADPKD organoid models using iPSCs. Organoids generated from iPSCs represent one of the most patient-specific models among organoid platforms, effectively mimicking disease states under controlled physiological conditions. The ongoing optimization of ADPKD organoid models is expected to enhance our understanding of the disease’s pathogenesis and facilitate the development of targeted therapies.

Another established preclinical model of polycystic kidney disease that has played an important role in the advancement of ADPKD research is the Han:SPRD Rat. In this Special Issue, Kofotolios et al. [10] review the utility of the Han:SPRD rat model, highlighting its phenotypic similarity to human ADPKD and its role in preclinical trials and as a tool for studying the pathogenesis of polycystic kidney disease and evaluating potential therapeutic interventions.
